# Findings of *DTI-p* maps in comparison with *T*_2_*/T*_2_*-FLAIR* to assess postoperative hyper-signal abnormal regions in patients with glioblastoma

**DOI:** 10.1186/s40644-018-0166-4

**Published:** 2018-09-18

**Authors:** Manijeh Beigi, Mojtaba Safari, Ahmad Ameri, Mohsen Shojaee Moghadam, Azim Arbabi, Morteza Tabatabaeefar, Hamidreza SalighehRad

**Affiliations:** 1 Quantitative MR Imaging and Spectroscopy Group, Research Center for Cellular and Molecular Imaging, Institute for Advanced Medical Imaging, Department of Medical Physics and Biomedical Engineering, Tehran University of Medical Sciences, Tehran, Iran; 2 Department of Energy Engineering, Sharif University of Technology, Tehran, Iran; 3 Department of Clinical Oncology, Shahid Beheshti University of Medical Science, Tehran, Iran; 4 Payambaran Imaging Center, Tehran, Iran; 5 Department of Medical Physics, Shahid Beheshti University of Medical Science, Tehran, Iran

**Keywords:** DTI, Glioblastoma, Isotropic map, Treatment planning

## Abstract

**Purpose:**

The aim of this study was to compare diffusion tensor imaging (DTI) isotropic map (*p-*map) with current radiographically (*T*_2_/*T*_2_*-FLAIR*) methods based on abnormal hyper-signal size and location of glioblastoma tumor using a semi-automatic approach.

**Materials and methods:**

Twenty-five patients with biopsy-proved diagnosis of glioblastoma participated in this study. *T*_2_, *T*_2_*-FLAIR* images and diffusion tensor imaging (DTI) were acquired 1 week before radiotherapy. Hyper-signal regions on *T*_2_, *T*_2_*-FLAIR* and DTI *p-*map were segmented by means of semi-automated segmentation. Manual segmentation was used as ground truth. Dice Scores (DS) were calculated for validation of semiautomatic method. Discordance Index (DI) and area difference percentage between the three above regions from the three modalities were calculated for each patient.

**Results:**

Area of abnormality in the *p*-map was smaller than the corresponding areas in the *T*_2_ and *T*_2_*-FLAIR* images in 17 patients; with mean difference percentage of 30 ± 0.15 and 35 ± 0.15, respectively. Abnormal region in the *p*-map was larger than the corresponding areas in the *T*_2_*-FLAIR* and *T*_2_ images in 4 patients; with mean difference percentage of 26 ± 0.17 and 29 ± 0.28, respectively. This region in the *p*-map was larger than the one in the *T*_2_ image and smaller than the one in the *T*_2_*-FLAIR* image in 3 patients; with mean difference percentage of 34 ± 0.08 and 27 ± 0.06, respectively. Lack of concordance was observed ranged from 0.214–0.772 for *T*_2_*-FLAIR/p*-map (average: 0.462 ± 0.18), 0.266–0.794 for *T*_2_ /*p-*map (average: 0.468 ± 0.13) and 0.123–0.776 for *T*_2_*/ T*_2_*-FLAIR* (average: 0.423 ± 0.2). These regions on three modalities were segmented using a semi-automatic segmentation method with over 86% sensitivity, 90% specificity and 89% dice score for three modalities.

**Conclusion:**

It is noted that *T*_2_*, T*_2_*-FLAIR* and DTI *p*-maps represent different but complementary information for delineation of glioblastoma tumor margins. Therefore, this study suggests DTI *p*-map modality as a candidate to improve target volume delineation based on conventional modalities, which needs further investigations with follow-up data to be confirmed.

## Introduction

Glioblastoma is the most aggressive brain tumor in adults. The current standard of care for patients with glioblastoma is maximal safe surgical de-bulking, followed by adjuvant radiotherapy with concurrent and adjuvant Temozolomide chemotherapy [1].

Diffuse and infiltrative growth of this tumor is a major determinant of poor prognosis. Inherent heterogeneity, unclear boundary and escaped invasive tumor cells are prominent aspects of glioblastoma, making accurate delineation of tumor boundary impossible using conventional MRI (cMRI). However, tumor delineation using cMRI is a necessary prerequisite step in diagnostic and therapeutic (monitoring treatment response) radiology in glioblastoma.

At present, treatment planning for glioblastoma tends to include the contrast-enhancing tumor on CT/*T*_1_-weighted MRI plus a 2 cm margin, or the *T*_2_-*FLAIR*/*T*_2_-weighted abnormality on the postoperative MRI scan plus a 1 cm margin [1]. Identifying the extension of abnormal region has been improved with recent evolutionary developments in MRI techniques [2].

Diffusion tensor imaging (DTI) is an advanced MRI method which is sensitive to infiltrated and disrupted white matter by tumor cells. Parametric DTI-maps can reveal peri-tumoral abnormalities that are not apparent on cMRI [3]. Price et al. have shown that isotropic (*p*) and anisotropic (*q*) components of water diffusion tensor are altered in peri-tumoral and gross tumor regions, respectively [3–5]. Tendency of glioblastoma to infiltrate along white matter tracts often leads to disease extension into peri-tumoral edema. Changes in white matter and edema architectures, as well as changes in cellularity cause an increase in isotropic diffusivity (essentially *p* parameter). Although detection of hyper–signal regions based on *T*_2_*/T*_2_*-FLAIR* images is the best marker for subclinical spread of the tumor, but it is not specific to the changes due to tumor infiltration [6–8]. Considering aforementioned issues, present study attempts to gain some insight into the spatial extension of postoperative hyper-signal region of glioblastoma on the three MRI modalities; *T*_2_, *T*_2_*-FLAIR* and DTI *p*-map using a semi-automatic segmentation method. Main aim of this study is to compare the three abovementioned segmented regions in size and location.

## Materials and method

### Patients selection and MRI data acquisition

Twenty-five patients (range 26 to 65 years) with a biopsy-proved diagnosis of glioblastoma were recruited after referral to our radiotherapy center for MR imaging. The institutional review board approved this study, and written informed consent was obtained from all subjects. Patients’ information is presented in Table 1.

**Table 1:** Patients Characteristics

Patient No.	Gender	Ages(Year)	Tumor Site
1	Male	30	Lt Frontal
2	Male	62	Rt Temporal
3	Male	30	Lt Temporal
4	Female	28	Rt Frontal
5	Female	54	Lt Frontal
6	Female	54	Lt Fronto-parietal
7	Female	26	Rt Frontal
8	Female	55	Lt Fronto-parietal
9	Male	54	Lt Frontal
10	Male	60	Rt Parietal
11	Male	65	Rt Parietal
12	Male	37	Rt Frontal
13	Male	53	Lt Temporal
14	Male	50	Rt Parietal
15	Female	53	Rt Parietal
16	Male	50	Lt Temporo-parietal
17	Male	54	Rt Parietal
18	Male	45	Rt Occipital
19	Female	36	Rt Frontal
20	Female	45	Rt Occipital
21	Female	50	Rt Occipital
22	Female	40	Rt Frontal
23	Male	52	Lt Parietal
24	Male	37	Rt Parietal
25	Female	36	Rt Parietal

MRI data acquisition was performed on a Siemens 1.5 T Avanto scanner (Siemens Healthcare) with a standard head coil. Conventional MRI protocols were as follows: *T*_2_-weighted fast spin-echo images (TR/TE = 3000/106 ms, FOV = 230 mm × 230 mm, Voxel size = 0.7 × 0.7 × 5.0 mm, slice thickness = 5 mm), *T*_2_*-FLAIR* images (TR/TE = 7000/93 ms, FOV = 230 mm × 230 mm, Voxel size = 0.9 × 0.9 × 5.0 mm, slice thickness = 5 mm), *T*_1_-weighted sequence (TR/TE = 1940/3 ms, FOV = 250 mm × 250 mm, Voxel size = 1 × 1 × 1 mm, slice thickness = 1 mm), and DTI (single-shot SE EPI sequence and diffusion gradients with two *b*-values (0, 1000 s/mm^2^) and 12 directions of gradient (TR/TE = 4500/101 ms, FOV = 240 mm × 240 mm, Voxel size = 1.8 × 1.8 × 3.0 mm, slice thickness = 3 mm, number of slices = 30)).

### DTI processing and registration

**Fig. 1:**
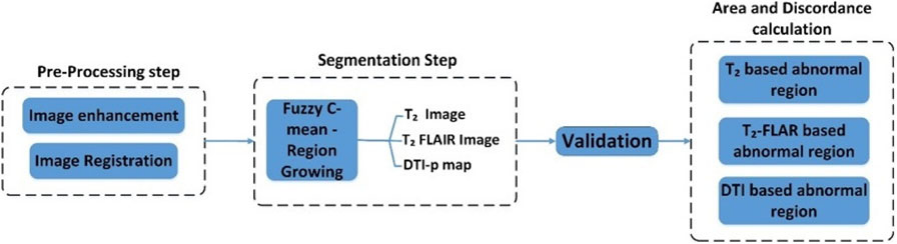
Block diagram of methodology

Block diagram for the entire procedure is shown in Fig. 1. DTI images were processed using Explore DTI (Version 4.8) software. After brain extraction, motion, eddy current and EPI corrections [9], three eigenvalues (ƛ_1_, ƛ_2_, ƛ_3_) and mean diffusivity (MD or D) were computed and used to calculate the isotropic component *p* ($p=\sqrt{3D}$) as previously described [5, 10]. After the *p*-map was obtained*, p*-map and *T*_2_ images were registered to the *T*_2_*-FLAIR* image as the reference image for each patient using a standard three dimensional (3D) cubic B-spline transformation with normalized mutual information cost function (SPM 12 software) [11] . Image enhancement was then applied on *p*-map for edge sharpening to improve results of the segmentation [12]. In addition, greyscale images were normalized to grey level values ranging from 0 to 1.

### Image segmentation

For each patient three MRI modalities were used for segmentation: *T*_2_, *T*_2_*-FLAIR* and *p-*map. A fuzzy C-means (FCM) clustering approach was implemented for segmentation of the images [13, 14]. FCM assumes that each pixel belongs to a cluster with constant intensities which is various in different tissue. Segmentation algorithm, based on fuzzy knowledge and region growing, separated the brain region into four classes in three MR modalities (*T*_2_, *T*_2_*-FLAIR* and *p*-map). *T*_2_ images were classified into four clusters: tissues with hyper-intensity values (necrosis, tumor hemorrhages and cysts), tissues with intermediate intensity (cerebrospinal fluid (CSF) and edema), tissues with hypo-intensity values (normal white and gray matter, scalp), and tissue with very low or zero intensity (skull, background). *T*_2_*-FLAIR* images also were classified into four classes: tissues with hyper-intensity values (necrosis, tumor hemorrhages and cysts), tissues with intermediate intensity (edema), tissues with hypo-intensity values (scalp, normal white and gray matter) and tissue with zero intensity (CSF, skull, background). Similarly, *p*-maps were classified into four clusters: tissues with high hyper-intensity values (tumor hemorrhages, cysts and CSF), tissues with intermediate intensity (edema), tissues with hypo-intensity values (normal white and gray matter), and regions with zero intensity (background). We used region growing method to group pixels together according to the rate of change of their intensities over a region. An arbitrary *seed pixel was chosen and* similar regions from seed point gradually coalesced into expanding regions. This whole process was continued until all pixels were grouped to one region.

The semiautomatic segmentation method was applied to each patient’s data. Segmentation results were validated based on manual expert’s segmentation. Hyper-signal abnormal regions on *T*_2*,*_ *T*_2_*-FLAIR* images, and obviously increased isotropy on the *p-*maps were manually segmented by a radiologist with 10 years of experience in neuro-oncology. They were visually evaluated and revised by another radiologist to obtain an accurate contour. Sensitivity, specificity and dice-score [15–17] were then calculated for alignment of the hyper-signal regions between each semi-automated and manual segmentation for each patient.

### Calculation of area of segmented region and discordance index

For the sake of comparison, the area of abnormal masks as segmented on *T*_2_, *T*_2_*-FLAIR* images and *p*-maps were calculated in square centimeter by multiplying all pixels’ sizes with the number of pixels. Three segmented regions were defined as follows: *A*_*T*_ (*T*_2_ derived abnormal region)*, A*_*F*_ (*T*_2_*-FLAIR* derived abnormal region), and *A*_*P*_ (*p-*map derived abnormal region). In addition, discordance index (*DI*), a measure of similarity in location was used for assessing agreement of locations of the three abnormal regions. This index was defined as the ratio of union of the two regions minus the intersection of the same two regions to the union of two regions, and as follows:

- $$ {DI}_{FP}=\frac{\left({A}_F\cup {A}_P\right)-\left({A}_F\cap {A}_P\right)}{A_F\cup {A}_P} $$
- $$ {DI}_{TP}=\frac{\left({A}_{T2}\cup {A}_P\right)-\left({A}_{T2}\cap {A}_P\right)}{A_{T2}\cup {A}_P} $$
- $$ {DI}_{TF}=\frac{\left({A}_{T2}\cup {A}_F\right)-\left({A}_{T2}\cap {A}_F\right)}{A_{T2}\cup {A}_T} $$

*DI*_*FP*_: discordance index between segmented regions on *T*_2_*-FLAIR* image and *p*-map*,*

*DI*_*TP*_: discordance index between segmented regions on *T*_2_ image and *p*-map,

*DI*_*TF*_: discordance index between segmented regions on *T*_2_ and *T*_2_*-FLAIR* images,

*DI* yields values between 0 (one region is perfectly similar or in agreement with another region) and 1 (two regions are completely apart). Higher score of *DI* means worse concordance between the two considered regions; low scores of *DI* mean better concordance.

## Results

### Evaluation of semiautomatic segmentation

**Fig. 2:**
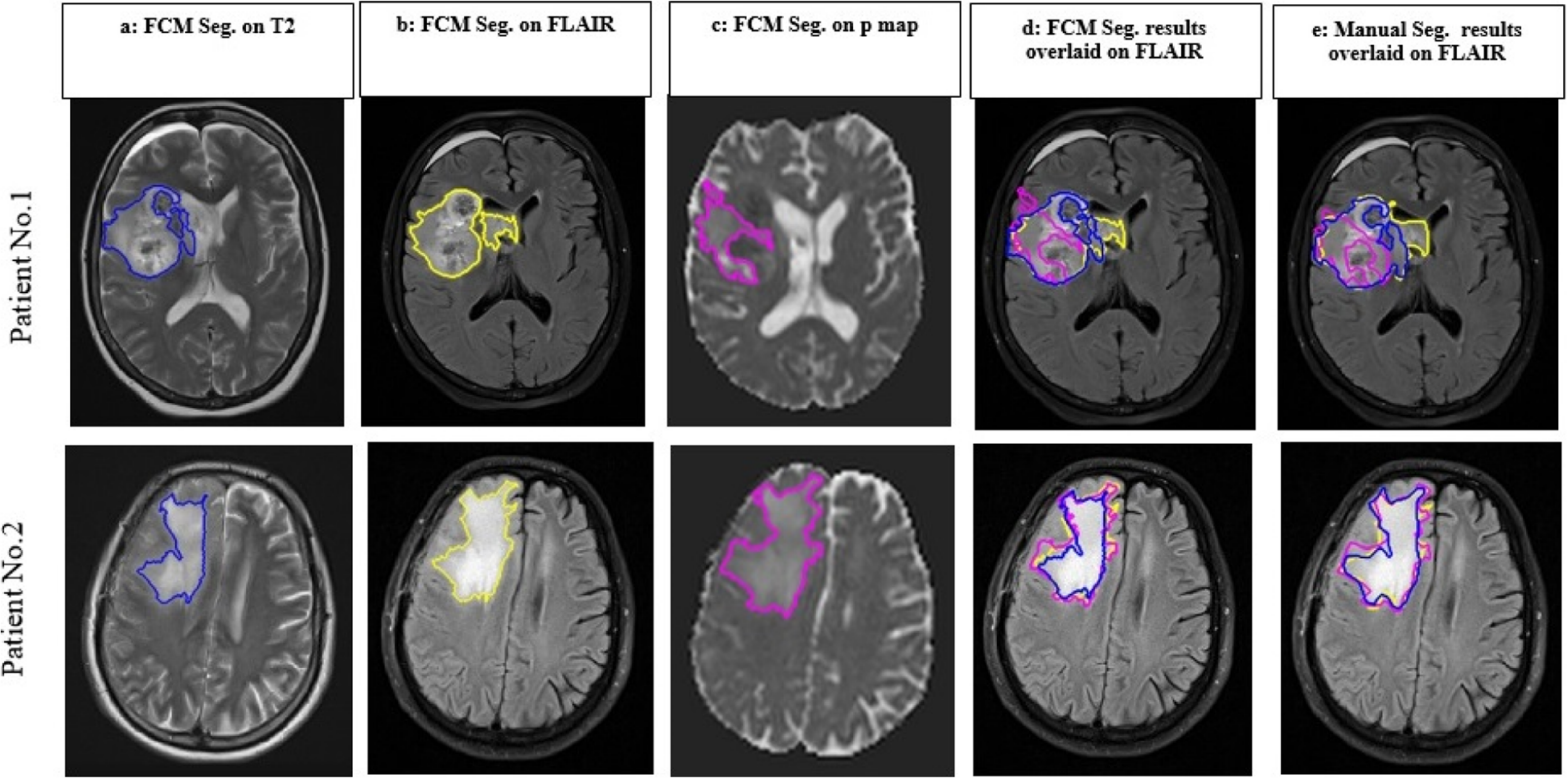
Results of segmentation on two patient’s data with glioblastoma who had undergone partial resection. Columns (**a-c**): FCM segmentation on (**a**) *T*_2_ (blue), (**b**) *T*_2_*-FLAIR* (yellow), (**c**) *p* maps (pink). (**d**) results of FCM segmentation overlaid on *T*_2_*-FLAIR* images, (**e**) Manual segmented region on three images overlaid on *T*_2_*-FLAIR*. **First row** represents the less agreement in location between *T*_2_*/T*_2_*-FLAIR* and *p*-map (**DI**_**TF**_ = 0.26, **DI**_**TP**_ = 0.57_,_ **DI**_**FP**_ = 0.65). **Second row** indicates the close similarity between three modalities (**DI**_**TF**_ = 0.18, **DI**_**TP**_ = 0.23_,_ **DI**_**FP**_ = 0.19)

Examples of the results of FCM– RG method and manual segmentation are presented for two patients in Fig. 2. Columns **a**, **b** and **c** demonstrate the results of FCM segmentation on *T*_2_*, T*_2_*-FLAIR* images and *p*-maps. Extracted hyper-signal abnormal regions from FCM and manual segmentation were overlaid on *T*_2_*-FLAIR* images that are shown in columns **d** and **e**, respectively**.** This figure shows that semi-automatic segmented regions visually correspond closely to the expert’s segmented ones. Three metrics as sensitivity, specificity and dice score were calculated for each patient and each modality. Average value of each metric over all patients are summarized in Table 2, showing that mean value of sensitivity and specificity are above 0.85 for each modality. Mean value for dice score over all patients between manual and semiautomatic contouring was 0.89 ± 0.08, 0.91 ± 0.05 and 0.92 ± 0.04 for segmented regions on *T*_2_, *T*_2_*-FLAIR* images and *p-*map, respectively. These values indicate that semi-automatic segmentation is matched well with the expert’s segmented regions.

**Table 2:** Evaluation of semiautomatic segmentation for each modality

	Sensitivity Mean(±SD)	Specificity Mean(±SD)	Dice Score Mean(±SD)
T_2_	0.86(±0.08)	0.92(±0.001)	0.89(±0.08)
T_2_-FLAIR	0.88(±0.07)	0.94(±0.04)	0.91 (±0.05)
DTI-p map	0.87(±0.05)	0.93(±0.01)	0.92 ± (0.04)

### Abnormal regions comparison in relation to size and discordance index

The area of segmented abnormal regions from *T*_2_*, T*_2_*-FLAIR* images and *p*-map are presented in Table 3.

A) Comparison of abnormality area in the *p*-map with the corresponding areas in the *T*_2_ and *T*_2_*-FLAIR* images:

- In 17 out of 25 patients, the abnormality area in the *p*-map was smaller than in the corresponding areas in the *T*_2_ and *T*_2_*-FLAIR* images (*A*_*P*_ < *A*_*T*2_, *A*_*P*_ < *A*_*F*_) with mean difference percentage of 30 ± 0.15 (min: 7%, max: 61%) and 35 ± 0.15 (min:13%, max:63%), respectively.
- In 4 out of 25 patients, the abnormality area in the *p*-map was larger than in the corresponding areas in the *T*_2_*-FLAIR* and *T*_2_ images (*A*_*P*_ > *A*_*F*_, *A*_*P*_ > *A*_*T*2_) with mean difference percentage of 26 ± 0.17 and 29 ± 0.28, respectively
- In 3 out of 25 patients, the abnormality area in the *p*-map was larger than the one in the *T*_2_ image and smaller than the one in the *T*_2_*-FLAIR* image (*A*_*T*2_ < *A*_*P*_ <*A*_*F*_) with mean difference percentage of 34 ± 0.08 and 27 ± 0.06, respectively.
- In 1 out of 25 patients, the regions on the three modalities were approximately equal in size (*A*_*P*_≃ *A*_*F*_ ≃ *A*_*T*2_) with mean difference value less than 5%.

B) Comparison of the abnormality in the *T*_2_ images with the corresponding area in the *T*_2_*-FLAIR* images:

- In 22 out of 25 patients, the abnormality area in the *T*_2_*-FLAIR* image was larger than the corresponding area in the *T*_2_ image with mean difference percentage of 27 ± 0. 29.
- In 3 out of 25 patients the abnormality area in the *T*_2_ image was larger than the corresponding area in the *T*_2_*-FLAIR* image with mean difference percentage of 15 ± 0.2.

**Table 3:** Obtained area and discordance indices between pathological region extracted from segmentation

Patient No.	Area(cm^2^)			Discordance Index		
	(P_T2_)	(P_F_)	(P_P_)	DI_TP_	DI_FP_	DI_TF_
1	15.07	18.04	12.35	0.609	0.481	0.672
2	7.18	12.67	8.89	0.411	0.304	0.457
3	13.95	14.43	13.99	0.296	0.340	0.187
4	12.32	14.23	17.06	0.472	0.506	0.598
5	7.24	14.98	10.12	0.577	0.472	0.429
6	32.52	34.58	17.98	0.572	0.583	0.393
7	35.97	33.19	24.19	0.476	0.591	0.367
8	18.02	19.27	22.15	0.258	0.321	0.225
9	6.84	8.85	3.95	0.329	0.379	0.347
10	5.16	6.10	2.95	0.589	0.640	0.228
11	23.05	25.87	21.76	0.266	0.215	0.128
12	11.84	13.67	7.50	0.508	0.575	0.395
13	13.00	13.61	10.02	0.457	0.686	0.453
14	4.75	7.97	2.98	0.480	0.761	0.569
15	8.21	9.52	3.14	0.794	0.772	0.731
16	29.09	23.94	18.84	0.433	0.230	0.336
17	8.39	8.52	9.85	0.300	0.254	0.240
18	12.25	12.01	9.12	0.315	0.214	0.178
19	14.85	9.59	7.32	0.495	0.615	0.658
20	6.12	6.18	4.72	0.512	0.248	0.123
21	3.08	2.52	4.26	0.375	0.411	0.261
22	5.19	7.35	4.45	0.482	0.522	0.714
23	9.62	9.88	8.14	0.430	0.472	0.430
24	8.59	11.05	12.5	0.721	0.239	0.689
25	10.13	12.25	6.62	0.549	0.738	0.776

Abbreviation: *P*_*T*_ *T*_*2*_ derived pathological region*, P*_*F*_ *T*_*2*_ *-FLAIR* derived pathological region, *P*_*P*_ *DTI-p* derived pathological region, *DT*_*FP*_ Discordance Index between *T*_*2*_*-FLAIR* and *p*-map, *DT*_*TP*_ Discordance Index between *T*_*2*_ and *p*-map, *DI*_*TF*_ Discordance Index between *T*_*2*_*-FLAIR* and *T*_*2*_

**Fig. 3:**
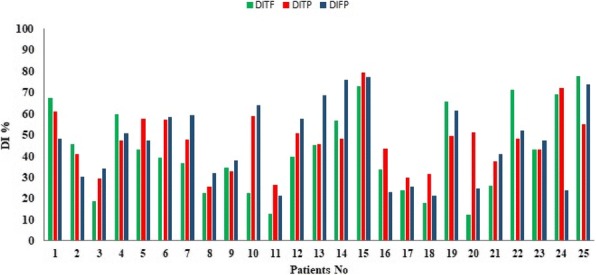
A graph showing the calculated DI% of abnormal regions extracted from three modalities. Green, Red and Blue indicate the of **DI**_**TF**_**, DI**_**TP,**_ **DI**_**FP**_ for twenty-five patients

In addition to calculation of area, there was a need to determine the degree of similarity in location of *A*_*P*,_ *A*_*F*_ and *A*_*T*2_. So, discordance indices (*DI*_*TP,*_ *DI*_*FP,*_ *DI*_*TF*_) were defined as written in method section. As reported in Table 3, there was a large range of discordance index between the three regions; *DI*_*TP,*_ *DI*_*FP*_ and *DI*_*TF*_ ranged from 0.266–0.794 (average: 0.468 ± 0.13), 0.214–0.772 (average: 0.462 ± 018) and 0.123–0.776 (average: 0.423 ± 0.2), respectively (Fig. 3).

Despite acquiring the results of differences in tumor extension and location (Table 3), intriguing findings were observed in some patients as follows:

In patient #21, a small hyper-signal abnormal region was seen in left temporal lobe on *T*_2_*-FLAIR* image and *p*-map that appeared normal on *T*_2_ images, while the tumoral region was detected in right occipital lobe.

In patient #11, a hyper-signal region was seen at the center of ventricles on *T*_2_*-FLAIR* image and *p*-map that was not detected on *T*_2_ image, while whole abnormal hyper-intense region was detected in right parietal lobe as listed in the Table 1.

## Discussion

Glioblastoma tumor predominantly infiltrates along white matter tracts and invades to surrounding edematous region [6, 18]. Previous studies on the behavior of glioblastoma suggest that DTI-derived tensor metrics can detect the integrity of white matter structures as a valid method without missing infiltrated brain areas [3, 5]. Hence, by calculating the isotropic (*p*) and anisotropic (*q*) metrics of diffusion tensor proposed by Pena et al. [10], it is possible to probe diseased brain parenchyma in the study of complex tumor such as glioblastoma. Price et al. have compared DTI-defined invasive and noninvasive regions using perfusion and magnetic resonance spectroscopy (MRS) [7]. They contoured *p* and *q* abnormalities to identify the invasive margin and then drew three regions of interest (ROIs) on *p*-invasive region (area of increased *p* and outside the area of reduced *q*), *p*-noninvasive region (outside of *p* abnormal region, in an area similar to the invasive ROI according to *T*_2_ image) and contralateral normal brain. Their results showed that there are significant differences in perfusion and MRS parameters between defined invasive and non-invasive regions based on the *p*-map. This study has clearly demonstrated that defined invasive and non-invasive regions based on *p/q*-maps look similar in appearance on *T*_*2*_ image but different in information content on the local environment. Furthermore, Price et al. in other studies have shown that increased DTI-isotropic component (*p*) around gross tumor indicates the infiltrating tumor margin [3, 5, 19]. These zones extend beyond abnormal areas on both enhanced *T*_1_- and *T*_2_-weighted MRI images. Four regions were selected on the abovementioned study; tumor, possible tumor infiltration near the tumor margin, edema and normal appearing contralateral white matter. From their spatial distribution of four regions in the *p: q* space, it can be observed that the healthy white matter has low *p* value and high *q* value with high variance. Tumor has high *p* value and very low *q* value. Edema has high *p* value and slightly lower *q* value than white matter and tumor margin with possible tumor infiltration demonstrate high *p* and low *q* values. As shown in these studies, it is proved that affected white matter tracts by tumor can be identified on DTI in four patterns categorized on the basis of isotropy and anisotropy (*p, q*) components. Accordingly, results of these studies demonstrate that the hyper-signal abnormal region on the *p*-map is appeared due to presence of either tumor or infiltrated white matter or edema.

On the other hand, and according to the current standards, clinical target volume concepts are based on either *T*_2_ or *T*_2_*-FLAIR* images to encompass possible microscopic disease. *T*_2_ and *T*_2_*-FLAIR* images are helpful for assessing non-enhancing tumor and edema extent but are not specific to changes due to tumor infiltration. Therefore, various MRI sequences reflect different properties of tissue, and no single imaging metric is currently sufficient to delineate the region of non-enhancing tumor. Consequently, we concluded that there is a need for further evaluation of extension of the hyper-signal regions on DTI *p*-map and *T*_2_*/T*_2_*-FLAIR* conventional images as a preliminary study. Thus, *p*-maps were considered beyond segmentation method for *T*_2_, *T*_2_*-FLAIR* images in FCM-RG semi-automatic segmentation procedure. Results of differences between size of abnormal regions on *T*_2_ and *T*_2_*-FLAIR* images (≃15%) for each patient show that using only one of these two structural techniques may not be adequate for delineation of boundary of the hyper-signal abnormal regions in radiotherapy planning. However, these images cannot differentiate between pure edema and tumor-infiltrated edema. Noticeble differences were found between the size and location of hyper-signal abnormalities on the *p*-map in comparison with *T*_2*/*_*T*_2_*-FLAIR* images. A large range of Discordance Index (*DI*) between the segmented abnormal regions on the *p-*map*/T*_2_ image and *p-*map*/T*_2_*-FLAIR* image in Table 3 represent that hyper-signal regions on three images were different not only in size but also in location. For example, for patient #3, results show that in spite of equality in abnormality’s size between three modalities, three regions are not completely concord to each other (*DI*_*TP*_ = 0.296_*,*_ *DI*_*FP*_ = 0.34 and *DI*_*TF*_ = 0.187). *T*_2_ or *T*_2_*-FLAIR* images only reveal partial tissue signatures of brain-tumor microenvironments. Furthermore, DTI *p*-map can identify diffusion signature of tissue and subtle white matter abnormalities. Therefore, *p*-map may be used to assist in delineation of whole abnormal hyper-signal regions in treatment planning of glioblastoma based on cMRI.

Main limitation of this study was DTI acquisition with only 12 directions and 2 *b*-value. Another important limitation was the lack of a follow-up imaging data to assess recurrence site in relation to three abnormal regions. Future work in this direction can include a larger prospective study based on a more patient population with follow-up imaging to investigate recurrence site.

## Conclusion

This study suggests that DTI *p*-map has the potential to improve target volume delineation based on *T*_2_ and *T*_2_*-FLIAR* modalities, but further investigation is needed to confirm it. Accurate manual segmentation of unclear boundary of abnormality on *p*-map is time-consuming and difficult, whilst the proposed segmentation procedure in this study results to decrease segmentation time. Therefore, this method might be a reliable way to segment hyper-signal regions on three modalities.

## Abbreviations

CSF: Cerebrospinal Fluid
CTV: Clinical Target Volume
DI: Discordance Index
DS: Dice Scores
DTI: Diffusion Tensor Imaging
FCM: Fuzzy C-Means
FLAIR: Fluid Attenuation Inversion Recovery
FOV: Field of View
MRI: Magnetic Resonance Imaging
TE: Time Echo
TR: Time Repetition
VE: Vasogenic Edema
